# Carcinosarcoma of the ovary: a case report and literature review

**DOI:** 10.3389/fonc.2023.1278300

**Published:** 2023-10-18

**Authors:** Jian Zheng, Cui Tang, Ping Liu, Hua Hao

**Affiliations:** ^1^ Department of Pathology, Yangpu Hospital, School of Medicine, Tongji University, Shanghai, China; ^2^ Department of Radiology, Yangpu Hospital, School of Medicine, Tongji University, Shanghai, China; ^3^ Department of Obstetrics and Gynecology, International Peace Maternity and Child Health Hospital, School of Medicine, Shanghai Jiao Tong University, Shanghai, China

**Keywords:** ovary, carcinosarcoma, clinicopathology, differential diagnosis, treatment, case report

## Abstract

**Objective:**

Carcinosarcoma of the ovary is a rare pathological type of ovarian cancer that is highly aggressive and occurs most frequently in the female reproductive tract at the site of the uterus. Herein, we explore the clinicopathological features, diagnosis, differential diagnosis, and treatment options for carcinosarcoma of the ovary.

**Methods:**

We analyzed the clinical data of a case of carcinosarcoma, observed its histological morphology and immunohistochemical characteristics, detected the homologous recombination repair deficiency gene mutation, and reviewed the relevant literature.

**Results:**

A 76-year-old menopausal woman visited our hospital because of abdominal distension, difficulty in urination, and constipation. Ultrasonography demonstrated abnormalities in the uterus and pelvic cavity, suggesting that the patient should undergo surgery. Immunohistochemical findings of carcinosarcoma of the right ovary were as follows: CK fraction (+), vimentin fraction (+), CK5/6 foci (+), p16 (+), p53 in approximately 70% (+), WT-1 foci (+), ER foci (+), PR part (+), Her-2 (1+), CK7 fraction (+), CK20 foci (+), CD99 fraction (+), CD10 fraction (+), CD56 foci (+), c-kit foci (+), SMA part (+), desmin foci (+), PD-L1 (-), SALL4 (-), OCT3/4 (-), p63 (-), p40 (-), D2-40 (-), inhibin (-), PLAP (-), CD30 (-), and Ki67 hotspot in approximately 80% (+). The patient underwent tumor cytoreduction and adjuvant chemotherapy. Currently, she is being followed up for 16 months and has a good general condition.

**Conclusion:**

The diagnosis of carcinosarcoma relies on histopathological examination and differentiation of carcinosarcoma from immature teratoma. The current therapeutic regimen for carcinosarcoma is still based on tumor cytoreduction and platinum-containing chemotherapy; research on targeted therapy is still in progress.

## Introduction

As known, 90% of ovarian cancers are of an epithelial cell type and comprise multiple histologic types, with various specific molecular changes, clinical behaviors, and treatment outcomes. The remaining 10% are non-epithelial ovarian cancers, which include mainly germ cell tumors, sex cord-stromal tumors, and some extremely rare tumors such as small cell carcinomas (1). Ovarian carcinosarcomas follow a distinct natural history (1). Carcinosarcoma of the ovary, also known as ovarian carcinosarcoma and malignant mixed mesodermal tumor of the ovary, is a rare pathological type of ovarian cancer that is highly aggressive and occurs most frequently in the female reproductive tract at the site of the uterus (2). Carcinosarcomas occurring in the ovary account for only 1–4% of all pathological types of ovarian cancer (3). They have an atypical clinical presentation, advanced stage at the time of diagnosis, poor prognosis, and recur within 1 year after the end of the initial treatment in most patients. Currently, there is a lack of a uniform, standardized, diagnostic and therapeutic protocol for carcinosarcoma of the ovary. Herein, we report a case of carcinosarcoma of the ovary and summarize its clinicopathological features and treatment options, in light of the relevant domestic and international literature, to improve the understanding of this tumor.

## Case description

### Clinical data

**Figure 1:**
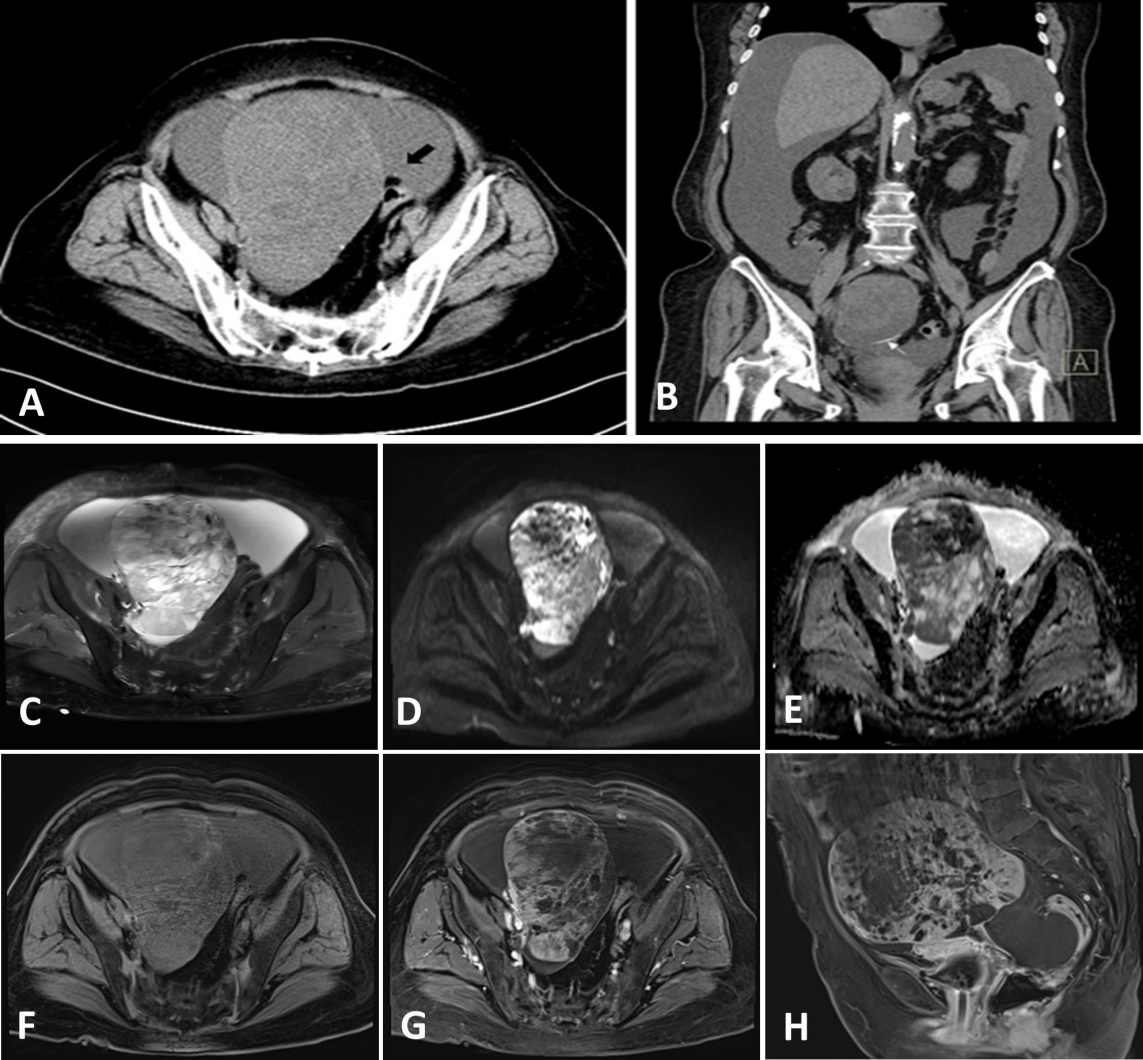
Computed tomography images **(A, B)** and enhanced magnetic resonance images **(C–H)** of the case.

The patient was a 76-year-old woman who experienced menopause for >20 years, without vaginal bleeding or other discomfort after menopause. Two weeks earlier, she experienced abdominal distension with difficulty in urination and defecation without obvious causes. On March 23, 2022, an abdominal ultrasonogram obtained outside the hospital showed an anterior uterus measuring 36 mm × 24 mm × 32 mm, endothelial thickness of 2 mm, cervical length of 25 mm, unequal echoes in the pelvic cavity, which measured 116 mm × 101 mm, unclear border, pelvic-free echogenic area of 110 mm × 96 mm, and the pelvic abdominal cavity in the echogenic area, with a depth of approximately 150 mm. The patient complained of abdominal distension, urinary difficulties, constipation, no abdominal pain, no irregular vaginal bleeding, and no increase in vaginal secretions. Therefore, she was admitted to our hospital as an emergency case for further investigation into the nature of the pelvic mass, as a malignant ovarian tumor was suspected. Physical examination revealed the following: bilateral adnexa not obvious to touch, pelvis could be touched a size of about 7 cm mass, activity check, no pressure pain. On admission, pelvic computed tomography (CT) showed a round, huge mass in the pelvic cavity, approximately 120 mm × 122 mm × 98 mm in size, with clear borders and uneven density. Low-density cystic necrosis was found inside. The CT value of the solid component of the lesion during plain scan was 24 HU. After enhancement, it appeared uneven. There was uniform, mild-to-moderate enhancement. The CT values in the arterial phase and venous phase were approximately 42 HU and 51 HU respectively. There was no obvious enhancement in the cystic necrosis area. It implied that the possible malignant pelvic tumor from the ovary may be an abdominopelvic cavity with a large amount of fluid or a small ascending colon diverticulum (**Figures 1A, B**). Enhanced magnetic resonance imaging (MRI) showed a huge mixed signal mass in the pelvic cavity. T1WI showed slightly low signal, T2WI showed obviously high and low mixed signal, T1WI-fs showed low mixed signal, T2WI-fs showed high and low mixed signal, and DWI (b=800) showed uneven signal. High signal, ADC value was about 1167×10^-6^ mm^2^/s; the size of the lesion was about 120 mm×122 mm×98 mm, with clear boundary and smooth edge, and the lesion was unevenly enhanced after enhancement. The left ovary was unclearly displayed, and the uterus was compressed. There was no obvious thickening of the endometrium. The signal was uniform, the junction zone was clear, and the muscle layer signal was uneven. There was no obvious enhancement of the endometrium and myometrium after enhancement. The cervical parenchyma showed multiple, abnormal, and round signal shadows of varying sizes, with low signal on T1WI and high signal on T2WI, and with clear boundaries and uniform signals. No obvious enhancement was seen after enhancement. The bladder was poorly filled and a urinary catheter was visible in the cavity. A large amount of fluid accumulation was seen in the abdominal and pelvic cavity. It revealed a huge malignant pelvic space, possibly originating from the ovary, a large amount of fluid in the abdominopelvic cavity, and multiple nasal cysts in the cervix (**Figures 1C–H**). The preliminary diagnoses were ovarian malignancy and pelvic-abdominal effusion. Laboratory tests revealed the following: CA125 level: 16.73 U/ml, CA199 level: <2 U/ml, and CEA level: 2.82 ng/ml, all of which were normal. However, human epididymis protein 4 (HE4) level was 283.5 pmol/L, which was higher than the normal level. The patient underwent surgery on March 31st, and the patient intraoperative observation revealed a large amount of bloody ascites (5000 ml) in the pelvic and abdominal cavity. A huge mass of about 12×11×10 cm, was seen in the right appendage, originating from the right ovary. The surface of the tumor was smooth without rupture, and no obvious tumor was found on the surface. The uterus was slightly smaller, about 4×3×2 cm, with a regular outline. There was dense adhesion between the bladder and the lower segment of the uterus. No obvious abnormality was found in the left appendage. The greater omentum was thickened and had a hard texture. The cancer had metastasized. The greater omentum was thickened in a pie shape, measuring about 13×12×6 cm. The surface of the liver was detected. Miliary metastasis was detected. The stomach and pelvic intestines had smooth serosal surfaces, the posterior leaf of the left broad ligament showed flaky thickening and frizzy peritoneal surfaces, and the rest of the pelvic and abdominal peritoneum was smooth. Based on the above intraoperative observations, the International Federation of Gynecology and Obstetrics (FIGO) stage of the patient was IIIc T3cNxM0.

### Pathological examination

**Figure 2:**
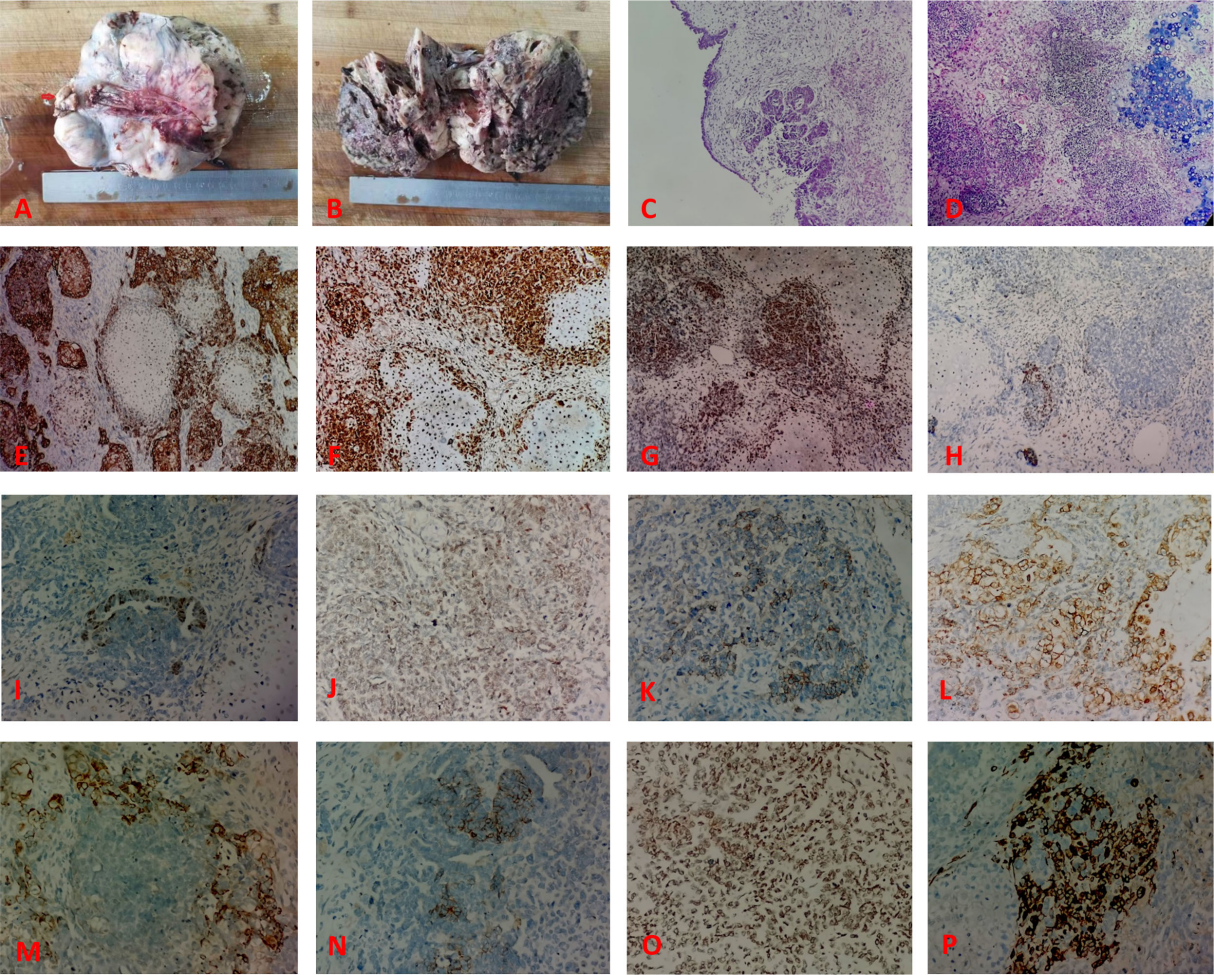
The pathological feature of the case. **(A)** The tumor is solid and nodular, with the umbilical end of the fallopian tube seen as a nodule on the surface (arrows). **(B)** The tumor is grayish dark red, solid, and has honeycombing in some areas. **(C)** Hematoxylin and eosin (HE) stain showing the epithelial component of the tumor (predominantly plasmacytoid carcinoma). **(D)** HE stain showing the poorly differentiated epithelial and mesenchymal components within the tumor (chondrosarcoma). Immunohistochemical findings: **(E)** epithelial component CK (+); **(F)** tumor cell p16 (+); **(G)** tumor cell p53 (+); **(H)** tumor cell WT-1 focal (+); **(I)** tumor cell ER foci (+); **(J)** PR part (+); **(K)** Her-2 (1+); **(L)** CK7 fraction (+); **(M)** CK20 foci (+); **(N)** c-kit foci (+); **(O)** SMA part (+); **(P)** desmin foci (+).

There was a clear indication for surgery, and open laparotomy was later performed. Results of the intraoperative rapid pathology report indicated a malignant tumor (right ovary), to be classified after paraffin sectioning and immunohistochemical examination. Collection and use of all specimens were approved by the Ethics Committee of Yangpu Hospital, School of Medicine, Tongji University. Abdominal tumor reduction, appendectomy, and partial salpingo-oophorectomy were performed. Postoperative gross pathological observation showed the following: right adnexa, soft, grayish dark red nodular mass measuring 14 cm × 12 cm × 8 cm with honeycombing in some areas; attached fallopian tube measuring 3 cm long, 5 cm in diameter; and nodular umbrella end measuring 2 cm × 2 cm × 1.5 cm with a medium texture (**Figures 2A, B**). Microscopically, the right ovarian mass showed plasma carcinoma with a poorly differentiated epithelial component; the mesenchymal component was chondrosarcoma (**Figures 2C, D**). The immunophenotyping of the right ovarian mass was as follows: ovarian tumor cells CK fraction (+) (**Figure 2E**), vimentin fraction (+), CK5/6 foci (+), p16 (+) (**Figure 2F**), p53 in approximately 70% (+) (**Figure 2G**), WT-1 foci (+) (**Figure 2H**), ER foci (+) (**Figure 2I**), PR part (+) (**Figure 2J**), Her-2 (1+) (**Figure 2K**), CK7 fraction (+) (**Figure 2L**), CK20 foci (+) (**Figure 2M**), CD99 fraction (+), CD10 fraction (+), CD56 foci (+), c-kit foci (+) (**Figure 2N**), SMA part (+) (**Figure 2O**), desmin foci (+) (**Figure 2P**), PD-L1 (-), SALL4 (-), OCT3/4 (-), p63 (-), p40 (-), D2-40 (-), inhibin (-), PLAP (-), CD30 (-), and Ki67 hotspot in approximately 80% (+). The pathological diagnosis was carcinosarcoma of the right ovary (the carcinoma component was high-grade plasmacytoid carcinoma, whereas the sarcomatoid component was chondrosarcoma). The peritoneal biopsy results indicated metastatic adenocarcinoma; carcinoma was observed in the greater omentum, left ovary, and left fallopian tube; there was no invasion of the tumor in the parietal uterus, vascular tissues of the right ovary or appendix, or vasculature and nerves. The results of the homologous recombination repair deficiency (HRD) gene test were as follows: HRD status was positive, the breast cancer susceptibility gene 1 (*BRCA1*) gene mutation was a variant of undetermined significance, and the tumor protein p53 (*TP53*) gene missense mutation with 86.15% mutation abundance, was a pathogenic variant.

### Treatment and follow-up

**Figure 3:**
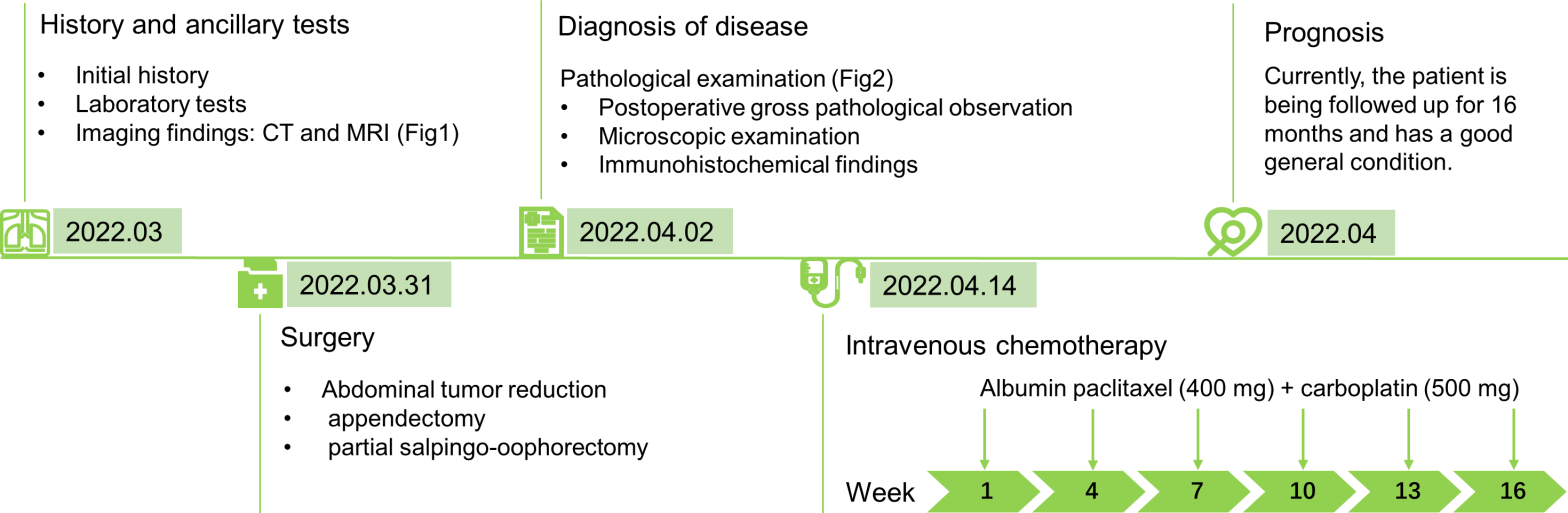
A timeline figure summarising the case diagnosis and treatment pathway.

To control the development of the disease and prolong the patient’s survival period, the proposed postoperative treatment was intravenous chemotherapy of albumin paclitaxel (400 mg) + carboplatin (500 mg) on day 1, every 21 days for six courses, and the first chemo treatment is April 14th (**Figure 3**). Currently, the patient is being followed up for 16 months and has a good general condition.

## Discussion

Carcinosarcoma is a highly malignant tumor with both epithelial and mesenchymal components, most often occurring in the uterus and extremely rarely in the ovaries (3). Herein, we identified a new case of carcinosarcoma and summarized the clinicopathological staging, treatment, and prognosis of the 58 previously reported cases (**Table 1**). Of the 59 cases reported to date, the age of onset was mostly 60–80 years in postmenopausal women (4). The risk factors associated with disease onset include obesity, childlessness, chronic estrogen use, and tamoxifen (5). Seventy-five percent of patients were reported to be at FIGO stages III–IV at the time of diagnosis, and 90% were at FIGO stages II–IV, with a significantly low survival rate (6).

**Table 1:** 58 cases of clinic data and prognosis data.

Age	Stage	Treatment	Metastasis	Follow-up months	Outcome
55	Ic	Surgery, CT	Yes	NA	NA
47	Ia	Surgery, CT	NA	71	AWD
62	IIIc	Surgery, CT	NA	59	AWD
66	IIIc	Surgery, CT	NA	11	DOD
78	IIIc	Surgery, CT	NA	7	DOD
54	IIIc	Surgery, CT	NA	44	DOD
83	IIIb	Surgery, CT	NA	11	Alive
55	IIIc	Surgery, CT	NA	35	Alive with disease
58	IIIc	Surgery, CT	NA	10	DOD
65	IIIc	Surgery, CT	NA	18	Alive with disease
82	IV	Surgery, CT	NA	26	AWR
80	IIc	Surgery, CT	NA	6	AWD
62	IIIb	Surgery, CT	NA	41	DOD
65	IV	Surgery, CT	NA	14	Alive
60	IIc	Surgery, CT	NA	12	Alive
46	Ic	Surgery, CT	NA	30	Alive
64	IIIc	Surgery, CT	NA	46	Alive with disease
52	IIIc	Surgery, CT	Yes	18	AWD
65	IIIc	Surgery, CT	NA	24	Alive
51	IIIa	Surgery, CT	NA	14	DOD
37	NA	Surgery	Yes	5 days	DOD
48	IV	Surgery, CT	Yes	9	DOD
37	IIc	Surgery, CT	Yes	13	Alive
64	IIIc	Surgery, CT	Yes	3	Alive
69	IV	Surgery	Yes	10 days	DOD
60	NA	Surgery, CT	Yes	4	Alive
NA	IIIc	Surgery, CT	NA	33	AWD
NA	IIIc	Surgery, CT	NA	8	AWD
NA	IIIc	Surgery, CT	NA	19	DOD
NA	Ic	Surgery, CT	NA	15	DOD
NA	IIIc	Surgery, CT	NA	4	DOD
NA	IIc	Surgery, CT	NA	24	Dead
NA	IIIc	Surgery, CT	NA	25	DOD
NA	IIIc	Surgery, CT	NA	110	AWD
NA	IIIc	Surgery, CT	NA	38	AWD
NA	IIIc	Surgery, CT	NA	18	DOD
NA	IIc	Surgery, CT	NA	13	DOD
NA	IIIc	Surgery, CT	NA	10	DOD
NA	IIIc	Surgery, CT	NA	23	DOD
50	NA	Surgery	Yes	2	DOD
55	IIc	Surgery, CT	NA	76	DOD
57	IIIc	Surgery, CT	NA	6	DOD
62	IIIc	Surgery, CT	NA	22	DOD
57	IIIc	Surgery, CT	NA	13	DOD
72	IIIc	Surgery, CT	NA	4	AWD
71	IIIc	Surgery, CT	NA	11	AWD
58	IIIc	Surgery, CT	NA	2	AWD
55	IV	Surgery, CT	NA	68	AWD
40	IIc	Surgery, CT	Yes	46	DOD
64	IV	Surgery, CT	NA	37	Dead
64	IIIc	Surgery, CT	NA	7	DOD
52	NA	Surgery, CT	NA	6	AWD
64	IIIa	Surgery, CT	Yes	26	AWD
75	IV	Surgery	Yes	NA	DOD
59	NA	Surgery	Yes	18	Dead
74	NA	Surgery	Yes	18	Alive
52	NA	Surgery, CT	Yes	12	AWD
78	IIIb	Surgery, CT	Yes	120	AWD

NA, data not available; CT, chemotherapy; AWD, alive without disease; DOD, dead of disease; AWR, alive with recurrence.

### Clinical manifestation

Carcinosarcoma is prevalent in postmenopausal elderly women. Our patient was 76 years old and was age compatible with other previously reported patients. Clinical manifestations of carcinosarcoma are similar to those of epithelial ovarian cancer, but aggressiveness is higher and the degree of malignancy is much higher. Carcinosarcoma is difficult to diagnose preoperatively, is not easily detected in the early stage, lacks specificity, has a rapid disease progression, is prone to metastasis, and most patients are in the late stage at the time of diagnosis. The main clinical manifestations include abdominal mass, abdominal distension, abdominal pain, ascites, occasional vaginal bleeding, and nonspecific gastrointestinal symptoms in some patients. A gynecological examination can reveal a pelvic mass with a large volume, irregular shape, unclear boundary, and poor activity. Gynecological ultrasonography can reveal a solid pelvic cystic mass. The present patient presented with abdominal distension and a pelvic mass.

However, the metastatic mechanism of carcinosarcoma is not fully understood. Direct spread, abdominal implantation, and lymphatic metastasis are considered important metastatic routes, similar to other malignant ovarian tumors. The rates of lymph node metastasis and vascular invasion in carcinosarcoma are high, and some researchers have reported that lymph node metastasis occurs in more than half of patients at the time of initial diagnosis (7–9). More than 90% of carcinosarcomas spread beyond the ovaries, and one-third of cases are associated with peritoneal effusion (7, 8). In our case, metastatic adenocarcinoma was observed from the peritoneal biopsy results, and carcinomatous involvement was observed in the greater omentum, left ovary, and left fallopian tube, which is consistent with findings in the literature.

### Histopathological features

There are several theories regarding the organizational origins of carcinosarcoma (3, 9). These theories include the (1) transformation theory, which suggests that the sarcoma component is transformed from the cancer component during the process of tumor derivation; (2) combinatorial theory, also known as the monoclonal origin theory, which suggests that the cancer and sarcoma components originate from a common pluripotent stem cell precursor that undergoes differentiation at the early stage of the tumor; and (3) collision theory, which suggests that the cancer and sarcoma components are independent of each other, originating from two different stem cells that ultimately collide to form the cancer/sarcoma. Currently, the theory of monoclonal origin is preferred. Additionally, some studies have reported the existence of gene mutations in carcinosarcoma, such as deletion of the breast cancer susceptibility gene 2 allele and *TP53* mutation (10, 11). In our patient, results of the HRD genetic test report showed that the patient was positive for HRD status, with a missense mutation in the *BRCA1* gene and a missense mutation in the *TP53* gene, which is located in exon 5 of the *TP53* gene; thereby, resulting in the substitution of amino acid 151 from proline to serine in the protein sequence encoded by the gene (10, 11). This mutation is considered pathogenic.

Microscopically, both epithelial and mesenchymal components were observed. The epithelial component can be an endometrioid or tubal epithelioid gland-like structure, squamous cell carcinoma, or clear cell carcinoma forming strips or nests; in this case, plasmacytoid carcinoma and poorly differentiated epithelial components were observed microscopically. The mesenchymal component can be endometrial mesenchymal sarcoma, smooth muscle sarcoma, chondrosarcoma, osteosarcoma, rhabdomyosarcoma, or liposarcoma, among which chondrosarcoma is the most common. The mesenchymal component in the present case was chondrosarcoma, which is in line with that in previous reports.

In addition, immunohistochemistry is valuable for the identification of different tissue components of carcinosarcoma (12, 13). In this case, immunohistochemistry findings were positive for CK and EMA in the epithelial component and diffusely positive for vimentin in the mesenchymal component; Ki-67 was also found to be positive in 60% of the cells (14). In our patient, the following findings were in accordance with those reported previously: CK fraction (+), vimentin fraction (+), CK5/6 foci (+), p16 (+), p53 in approximately 70% (+), WT-1 foci (+), ER foci (+), PR part (+), Her-2 (1+), CK7 fraction (+), CK20 foci (+), CD99 fraction (+), CD10 fraction (+), CD56 foci (+), c-kit foci (+), SMA part (+), desmin foci (+), PD-L1 (-), SALL4 (-), OCT3/4 (-), p63 (-), p40 (-), D2-40 (-), inhibin (-), PLAP (-), CD30 (-), and Ki67 hotspot in approximately 80% (+).

### Imaging

The CT manifestation of carcinosarcoma is commonly a cystic-solid mixed density mass, mostly located unilaterally, more on the right side than on the left side, with multiple small cystic cavities of varying sizes in the capsule, varying thicknesses of the wall, and the solid part of the tumor is flocculent or nodular shape. The tumor parenchyma is mostly in the form of hypodensities on plain CT, and the area of necrotic cystic degeneration is in the form of lower densities. CT can accurately locate the carcinosarcoma and provide information on the size and shape of the lesion, internal structure, and growth characteristics. It is of great value to observe whether there is invasion of neighboring tissues and organs and whether there are lymph nodes and distant metastases. The CT examination of this patient showed a round mass in the pelvis, approximately 93 mm × 118 mm, with a clear boundary, uneven density, CT value of approximately 24 HU, and calcification at some edges; there was no obvious obstruction and dilatation of the lower abdomen and pelvic intestines; the bladder was well filled; the bladder wall was smooth and non-thick; there was no obvious abnormality in the bladder lumen; there were no obvious enlarged lymph nodes in the bladder; the abdominopelvic cavity was filled with a large amount of fluid; and a small cystic pouch protruding shadow was seen in the ascending colon. However, CT image staging and surgical pathology staging could not be matched. When a large amount of peritoneal fluid is present on CT images, it does not clearly show peritoneal implantation and regional lymph node metastasis. In particular, the sensitivity of discovering small nodes is poor, so pathology is still the gold standard for confirming the diagnosis of carcinosarcoma. Nevertheless, CT plays an important role in the observation of invasion of the tumor’s neighboring organs and tissues, the presence or absence of pelvic effusion, and the metastasis of the peritoneum and lymph nodes, which provides an important basis for the clinical staging of the tumor.

### Diagnosis and differential diagnosis

The clinical manifestations, imaging manifestations, and serological indices of carcinosarcoma are nonspecific and almost indistinguishable from other types of malignant ovarian tumors; therefore, preoperative diagnosis is difficult. Hence, diagnosis of carcinosarcoma requires a combination of clinical and pathological findings. In this case, the pathological diagnosis was an carcinosarcoma of the right ovary. The most important differential diagnosis of Carcinosarcoma is immature teratoma, which is distinguished by two factors. (1) Age of disease onset: Carcinosarcoma is almost always seen in postmenopausal women, whereas immature teratomas are more commonly seen in postmenopausal women, children, and young adults. (2) Pathomorphology: Carcinosarcoma is a simpler mixture of multiple malignant epithelia and mesenchyme, with immature embryonic organ-like structural changes without differentiation to the tertiary germ layer, and usually lacks the neural and germ cell components of teratomas, whereas immature teratomas tend to have embryonic neural ectodermal differentiation, such as neural tubes, which is important and can be distinguished from other teratomas. This information is important for differentiation.

### Treatment

The current treatment for carcinosarcoma involves a combination of surgical procedures, often followed by adjuvant chemotherapy. Most retrospective studies have affirmed the role of tumor cytoreduction in the treatment of ovarian carcinosarcoma, and better survival has been achieved by optimal tumor reduction. Satisfactory tumor cytoreduction was defined as a maximum residual focus of <1 cm in diameter after surgery. Doo et al. (2) reported that in 51 patients after tumor cytoreduction, the median durations of progression-free survival (PFS) of the three groups with no visible residual foci (n = 18), visible residual foci with a maximum diameter of ≤1 cm (n = 20), and >1 cm (n = 13) were 29, 21, and 2 months, respectively (P = 0.036); and the median durations of overall survival were 57, 32, and 11 months, respectively, with statistically significant differences (P < 0.05). Therefore, satisfactory tumor cytoreduction may improve patient prognosis, and residual lesions should be minimized during surgery to prolong patient survival.

Because of the lack of clinical studies with large datasets, the efficacy of first-line chemotherapy regimens is inconclusive, and platinum-based combination chemotherapy is currently used. Brackmann et al. (15) retrospectively analyzed 31 patients diagnosed with ovarian or primary peritoneal carcinosarcoma, and patients treated with carboplatin/paclitaxel had a significantly longer PFS than those receiving isocyclophosphamide/paclitaxel (17.8 versus 8.0 months). However, Yalcin et al. (16) evaluated the effect of satisfactory tumor cytoreduction followed by adjuvant paclitaxel in combination with platinum-based chemotherapy, on survival outcomes in 54 patients with ovarian carcinosarcoma and 108 patients with epithelial carcinoma of the ovary, both of whom underwent satisfactory tumor cytoreduction. They showed that treating patients with carcinosarcoma of the ovary and epithelial carcinoma of the ovary with the same regimen resulted in no significant difference in PFS durations of 29 and 27 months, respectively. Considering the present patient’s condition, intravenous chemotherapy was administered on day 1 for 21 days.

Owing to the lack of therapeutic efficacy, several studies on biologically targeted therapies to improve efficacy are underway. Zhu et al. (17) detected the expression of programmed cell death ligand 1 (PD-L1) in 19 cases of carcinosarcoma and found that there was positive expression of PD-L1 in 52.6% of the cancer component and 47.4% of the sarcoma component; those with negative expression of PD-L1 in the sarcoma component had a significantly higher survival rate than those with positive expression (P = 0.036). The PD-1/PD-L1 signaling pathway may be a new target for tailoring immunotherapy. Vascular endothelial growth factor expression has also been reported in ovarian and uterine cancer sarcomas and is associated with tumor progression and poor prognosis (18). Tang et al. (19) found that a murine sarcoma virus oncogene (*KRAS*) mutation and p53 deletion in mouse ovarian epithelial cells can induce carcinosarcoma, the epithelial component of which is mainly endometrioid carcinoma, and that the tumor metastasizes quickly, with a significantly higher risk of death. We reviewed 58 cases, and the clinic data and prognosis data are shown in **Table 1** (20–42). The maximum survival of 58 cases was 120 months; the disease-free survival of our case was 16 months, better than that of most of the cases. Results of the HRD genetic test report showed that our patient was positive for HRD status, with a missense mutation in the *BRCA1* gene and a missense mutation in the *TP53* gene, located in exon 5 of the *TP53* gene, resulting in the substitution of amino acid from proline to serine in its protein sequence. These germline mutations represent the most potent known genetic risk factors for epithelial ovarian cancers and are detected in 6–15% of women diagnosed with this condition. Knowledge of a patient’s BRCA1/2 status can play a pivotal role in counseling, particularly in predicting their expected survival. Notably, BRCA1/2 carriers with epithelial ovarian cancers exhibit a more favorable response to platinum-based chemotherapies, resulting in enhanced survival rates. Determining the prevalence of BRCA1 and BRCA2 mutations in ovarian carcinosarcomas poses challenges. Nevertheless, compelling evidence suggests that BRCA-wild type tumors can also display a BRCA-like phenotype, often referred to as “BRCAness”. Ovarian carcinosarcomas harboring loss-of-function mutations in homologous recombination genes may respond therapeutically to PARP inhibition (43). So, the medication recommendation suggests olaparib and niraparib as sensitive drugs and rucaparib, fluzoparib, pamiparib, and talazoparib as potentially beneficial drugs.

### Prognosis

Carcinosarcoma is far more malignant than are tumors of the uterus and fallopian tubes; there exists a clear relationship between the prognosis of patients and the type of pathology, clinical stage, cancer antigen 125 level, size of the residual tumor after surgery, and chemotherapy regimen. Most patients have a short survival period, with a mean survival of 11–12 months. Patients with chondrosarcoma-containing components have a longer survival period than those without chondrosarcoma-containing components. In our case of carcinosarcoma containing a chondrosarcoma component, the patient has a good general condition and is still being followed.

In summary, the incidence of ovarian carcinosarcoma is low, the symptoms are atypical, there are no specific serological indexes and imaging manifestations, and the disease progresses rapidly with poor prognosis. Therefore, in practice, it is necessary to pay careful attention to the diagnosis based on the combination of clinical and pathological findings for cystic solid tumors of the ovary, and it is necessary to perform comprehensive and multi-location sampling to provide a sufficient basis for diagnosis and differentiation from immature teratoma. This may help improve the early diagnosis rate and reduce the morbidity and mortality rates. Additionally, the best therapeutic option is still uncertain, and targeted therapy is still being researched. If suitable therapeutic targets can be found, the prognosis of patients will be greatly improved. Lastly, as carcinosarcoma generally develops at an older age, new treatment options and associated toxic effects should be considered in future studies, as they may not be well tolerated in the older patient population. Treatment options with fewer toxic side effects and better efficacy should be actively explored, which will in turn improve the prognosis of carcinosarcoma.

## Data availability statement

The original contributions presented in the study are included in the article/supplementary material. Further inquiries can be directed to the corresponding author.

## Ethics statement

Written informed consent was obtained from the individual(s) for the publication of any potentially identifiable images or data included in this article. Written informed consent was obtained from the participant/patient(s) for the publication of this case report.

## Author contributions

JZ: Conceptualization, Investigation, Writing – original draft. CT: Data curation, Formal Analysis, Resources, Writing – original draft. PL: Data curation, Resources, Writing – original draft. HH: Conceptualization, Funding acquisition, Investigation, Project administration, Resources, Supervision, Writing – review & editing.
